# Porcine endogenous retroviruses PERV A and A/C recombinant are insensitive to a range of divergent mammalian TRIM5*α* proteins including human TRIM5*α*

**DOI:** 10.1099/vir.0.007377-0

**Published:** 2009-03

**Authors:** Andrew Wood, Benjamin L. J. Webb, Birke Bartosch, Torsten Schaller, Yasuhiro Takeuchi, Greg J. Towers

**Affiliations:** 1 MRC Centre for Medical Molecular Virology, Division of Infection and Immunity, University College London, 46 Cleveland Street, London W1T 4JF, UK; 2 Université de Lyon, (UCB-Lyon1), IFR128, Lyon, F-69007, France; INSERM, U758, Lyon, F-69007, France; Ecole Normale Supérieure de Lyon, Lyon, F-69007, France

## Abstract

The potential risk of cross-species transmission of porcine endogenous retroviruses (PERV) to humans has slowed the development of xenotransplantation, using pigs as organ donors. Here, we show that PERVs are insensitive to restriction by divergent TRIM5*α* molecules despite the fact that they strongly restrict a variety of divergent lentiviruses. We also show that the human PERV A/C recombinant clone 14/220 reverse transcribes with increased efficiency in human cells, leading to significantly higher infectivity. We conclude that xenotransplantation studies should consider the danger of highly infectious TRIM5*α*-insensitive human-tropic PERV recombinants.

## INTRODUCTION

Pig to human xenotransplantation has been proposed as a way to alleviate the shortage of human donor organs used to treat a wide range of important medical conditions. However, it has been suggested that this process might lead to zoonosis of pathogens from the porcine organ to the human host, particularly after immunosuppression (Stoye & Coffin, 1995; Stoye *et al.*, 1998). Indeed, expression of porcine endogenous retroviruses (PERV) has been demonstrated in pigs and their presence in the germ line will make them difficult, if not impossible, to eliminate (Le Tissier *et al.*, 1997; Patience *et al.*, 2001). The notion that gammaretroviruses can be zoonotic is supported by the presence of highly related gammaretroviruses in unrelated species such as gibbons (gibbon ape leukemia virus) and koalas (koala retrovirus) (Hanger *et al.*, 2000; Tarlinton *et al.*, 2006). Their high degree of relatedness is interpreted as demonstrating zoonosis to gibbons and koalas, possibly from mice (Lieber *et al.*, 1975). Importantly, these viruses are pathogenic, causing leukaemia in both gibbons and koalas. As PERV sequences are closely related to these viruses, it is reasonable to suppose that in the right circumstances they too would be zoonotic, and potentially pathogenic if transmitted to humans.

TRIM5*α* has recently emerged as an important mediator of antiretroviral innate immunity in mammals. TRIM5*α* blocks retroviral infection in a species-specific way, for example human immunodeficiency virus type 1 (HIV-1) is restricted by TRIM5*α* from Old World monkeys, but not by human TRIM5*α* (Hatziioannou *et al.*, 2004; Stremlau *et al.*, 2004; Yap *et al.*, 2004). Human TRIM5*α* restricts infection by equine infectious anemia virus (EIAV) and the murine leukemia virus (MLV-N) (Hatziioannou *et al.*, 2004; Keckesova *et al.*, 2004; Yap *et al.*, 2004). TRIM5*α* encodes RING, B-box and coiled-coil domains, comprising a tripartite motif, as well as a C-terminal B30.2 domain, which determines antiviral specificity, and appears to interact directly with the incoming viral capsid (Stremlau *et al.*, 2006a). TRIM5*α* is thought to mediate an important barrier to zoonotic transmission of retroviruses by preventing replication early in the viral life cycle, usually before significant reverse transcription. The antiviral mechanism of TRIM5*α* remains incompletely characterized but appears to involve viral uncoating as well as recruitment to the proteasome (Anderson *et al.*, 2006; Stremlau *et al.*, 2006a; Wu *et al.*, 2006; reviewed by Towers, 2007). As PERVs have been suggested as a possible source of zoonotic infection after pig to human xenotransplantation we sought to examine the sensitivity of PERV isolates to restriction by mammalian TRIM5*α* molecules. Here, we show that two PERV isolates, prototypic PERV A PK (Bartosch *et al.*, 2002) and a high-titre PERV A/C recombinant PERV-A 14/220 (Bartosch *et al.*, 2004), are insensitive to restriction by divergent mammalian TRIM5*α* proteins. Furthermore, we show that the higher infectivity of the PERV A/C recombinant gag–pol is due to increased efficiency of reverse transcription.

## METHODS

### Cell lines and viral titrations.

Feline cells expressing TRIM5*α* proteins from human, African green monkey (Keckesova *et al.*, 2004), rhesus macaque, squirrel monkey (Ylinen *et al.*, 2005), cattle (Ylinen *et al.*, 2006) and rabbit (Schaller *et al.*, 2007) have been described previously.

PERV A and A/C recombinant gag–pol expression vectors were generated by replacing the G ORF with PERV gag–pol derived from PERV A (GenBank accession no. AY099323) and/or PERV A/C 14/220 (GenBank accession no. AY570980) at the *Bam*HI site in phCMV-G, by PCR (Bartosch *et al.*, 2003). Vesicular stomatitis virus glycoprotein (VSV-G) pseudotyped viral vectors derived from HIV-1 (Bainbridge *et al.*, 2001; Zufferey *et al.*, 1997), MLV (Bock *et al.*, 2000) and simian immunodeficiency virus mac (SIVmac) (Negre *et al.*, 2000) encoding green fluorescent protein (GFP) have been described elsewhere and were prepared by transfection of 293T cells as described previously (Besnier *et al.*, 2002). PERV GFP-encoding vectors were prepared similarly using a GFP-encoding genome derived from MLV (Naviaux *et al.*, 1996). PERV A/C gag–pol expression plasmids encoding PERV A protease, reverse transcriptase or integrase were constructed using the unique *Bcl*I site at the protease–reverse transcriptase junction or the unique *Hpa*I site at the reverse transcriptase–integrase junction.

### Western blot analysis.

A 1 ml sample of each viral supernatant, or supernatant from untransfected cells, was pelleted (123 000 ***g***, 90 min) and resuspended in 30 μl Laemmli buffer. A volume of 10 μl was subjected to PAGE, blotted and detected using a rabbit anti-PERV polyclonal antibody (Bartosch *et al.*, 2002) (1 : 1000) and an anti-rabbit horseradish peroxidase linked antibody (1 : 3000; GE Healthcare).

### Quantitative PCR (QPCR) to measure products of reverse transcription.

TaqMan QPCR to measure viral DNA synthesis was performed using primer/probe sequences specific to GFP as described previously (Passerini *et al.*, 2006). Cells (4×10^5^) were infected in six-well plates in triplicate with equivalent doses of virus treated with DNase (70 U ml^−1^ for 2 h; Promega). Six hours after infection, total DNA was extracted from two samples using a QiaAmp DNA extraction kit (Qiagen). The third sample was subjected to FACS analysis 48 h after infection to enumerate infected cells. DNA (100 ng) was subjected to TaqMan QPCR as described previously (Towers *et al.*, 1999). Absolute numbers of GFP DNA per PCR were determined by reference to a standard curve. The number of GFP molecules per 100 ng total DNA were plotted. As a negative control for plasmid contamination of the viral inoculum, cells were infected with virus that had been boiled for 5 min. QPCR was then performed as described above.

## RESULTS

### PERVs are insensitive to divergent TRIM5*α* proteins

**Fig. 1. f1:**
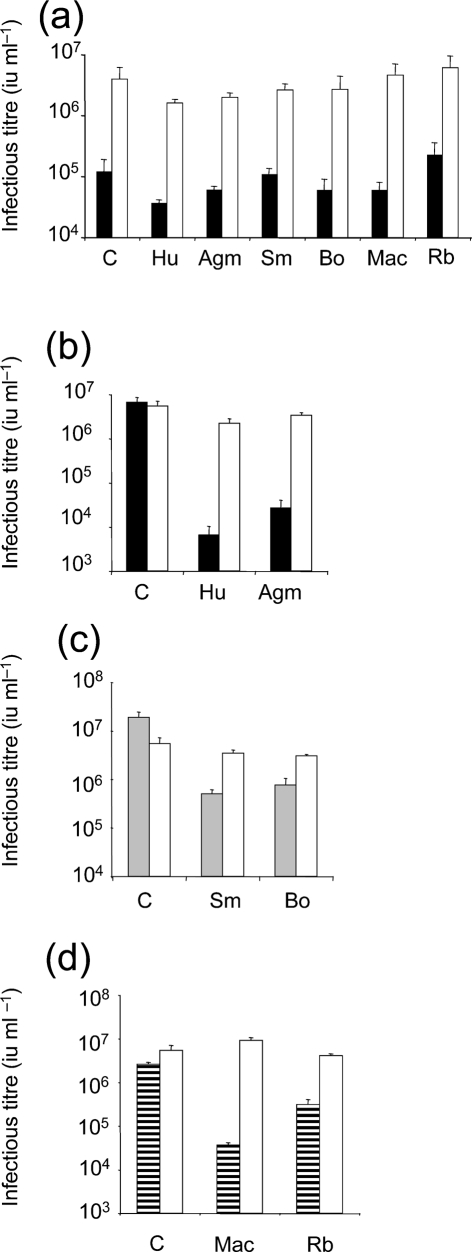
PERV are insensitive to divergent TRIM5*α* proteins. (a) GFP-encoding VSV-G pseudotypes of PERV A (black bars) or PERV A/C recombinant (white bars) were titrated on CRFK cells expressing TRIM5*α* from human (Hu), African green monkey (Agm), squirrel monkey (Sm), cattle (Bo), rhesus macaque (Mac) or rabbit (Rb) or unmodified CRFK cells as a control (C). GFP-encoding VSV-G pseudotypes of MLV-N (black bars) or MLV-B (white bars) SIVmac (grey bars) or HIV-1 (striped bars) were titrated onto CRFK cells expressing TRIM5*α* from human (Hu) or African green monkey (Agm) (b), squirrel monkey (Sm) or cattle (Bo) (c) or rhesus macaque (Mac) or rabbit (Rb) (d) or unmodified CRFK cells (C) as a control. Titres are expressed as infectious units ml^−1^ (iu ml^−1^). Errors bars indicate sd derived from two experiments performed with independent virus stocks.

Replication-competent PERV sequences have been divided into three classes, PERV A, B and C, according to their envelope sequences (Patience *et al.*, 1997; Takeuchi *et al.*, 1998). PERVs A and B have been shown to be able to infect human cells, and, importantly, naturally occurring high-titre PERV A/C recombinants have been described with the ability to replicate to high titres *in vitro* (Le Tissier *et al.*, 1997; Oldmixon *et al.*, 2002; Patience *et al.*, 1997; Takeuchi *et al.*, 1998; Wilson *et al.*, 2000). In order to test PERV sensitivity to TRIM5*α* we made gag–pol expression vectors for the prototypic PERV A PK and the PERV A/C 14/220 recombinant and used these plasmids to make VSV-G pseudotyped vectors packaging GFP-encoding MLV genomes as described previously (Besnier *et al.*, 2002). These viruses were then titrated onto permissive feline Crandall-Reese feline kidney (CRFK) cells expressing TRIM5*α* proteins from human, African green monkey, rhesus macaque, squirrel monkey, rabbit or cattle or unmodified CRFK cells as a negative control, as described previously (Keckesova *et al.*, 2004; Schaller *et al.*, 2007; Ylinen *et al.*, 2005, 2006) (Fig. 1a[Fig f1]). Forty-eight hours after exposure to PERV, infected cells were enumerated by counting fluorescent, GFP-expressing cells, by FACS. Infectious titres of each virus were then calculated and plotted (Fig. 1). Remarkably, the two PERVs were largely insensitive to all TRIM5*α*s tested. The strongest restriction was by human TRIM5*α*, but this led to only around a threefold reduction in infectivity.

As a positive control for the expression of each TRIM5*α*, titres of restriction-sensitive VSV-G pseudotyped GFP-encoding vectors were measured as above. In each case the viruses were selected for sensitivity to the TRIM5*α* in question. MLV-N infectivity was reduced by two to three orders of magnitude by expression of either human or African green monkey TRIM5*α* as described elsewhere (Hatziioannou *et al.*, 2004; Keckesova *et al.*, 2004; Perron *et al.*, 2004; Yap *et al.*, 2004) (Fig. 1b[Fig f1]). SIVmac infectivity was reduced by one to two orders of magnitude by expression of squirrel monkey or bovine TRIM5*α* (Fig. 1c[Fig f1]) and HIV-1 infectivity was reduced by expression of rabbit or rhesus TRIM5*α*, as described previously (Si *et al.*, 2006; Song *et al.*, 2005; Stremlau *et al.*, 2004; Ylinen *et al.*, 2005, 2006) (Fig. 1d[Fig f1]). MLV-B infectivity acted as a TRIM5*α*-insensitive control and was not affected by expression of any of the TRIM5*α* genes, as has been described previously (Hatziioannou *et al.*, 2004; Keckesova *et al.*, 2004; Perron *et al.*, 2004; Schaller *et al.*, 2007; Yap *et al.*, 2004; Ylinen *et al.*, 2005, 2006) (Fig. 1b–d[Fig f1]).

### PERV A and PERV A/C VSV-G pseudotypes contain similar amounts of p30 capsid protein

**Fig. 2. f2:**
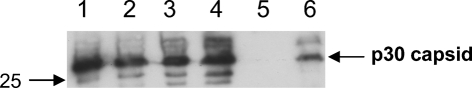
PERV A and PERV A/C VSV-G pseudotypes contain similar amounts of p30 capsid protein. Two stocks of PERV A (lanes 1 and 2) and two stocks of PERV A/C (lanes 3 and 4) were Western blotted to detect capsid protein. Supernatant from untransfected 293T cells was blotted as a negative control (lane 5) and a total extract of PERV A/C 14/220-infected 293T cells was blotted as a positive control (lane 6). The band representing p30 capsid is marked with an arrow on the right and the position of the 25 kDa size marker (Precision Plus Protein Standards; Bio-Rad) is shown on the left.

Fig. 1 demonstrates that the titre of the VSV-G pseudotyped PERV A/C recombinant is significantly higher than that of the VSV-G pseudotyped PERV A. This is consistent with previous observations made comparing PERV A and PERV A/C viral replication *in vitro* (Bartosch *et al.*, 2004). In order to control for the dose of the two viruses we compared the amounts of PERV capsid in the virus stocks by Western blot analysis using a rabbit anti-PERV polyclonal antibody to detect PERV gag (Fig. 2) (Bartosch *et al.*, 2002). The blot shows that the PERV A stocks (lanes 1 and 2) and PERV A/C stocks (lanes 3 and 4) have similar amounts of p30 capsid, demonstrating that each contained a similar concentration of virions. Supernatant from untransfected 293T cells and PERV A/C 14/220-infected cell lysate were blotted as controls (lanes 5 and 6, respectively).

### PERV A/C has more efficient reverse transcription than PERV A

**Fig. 3. f3:**
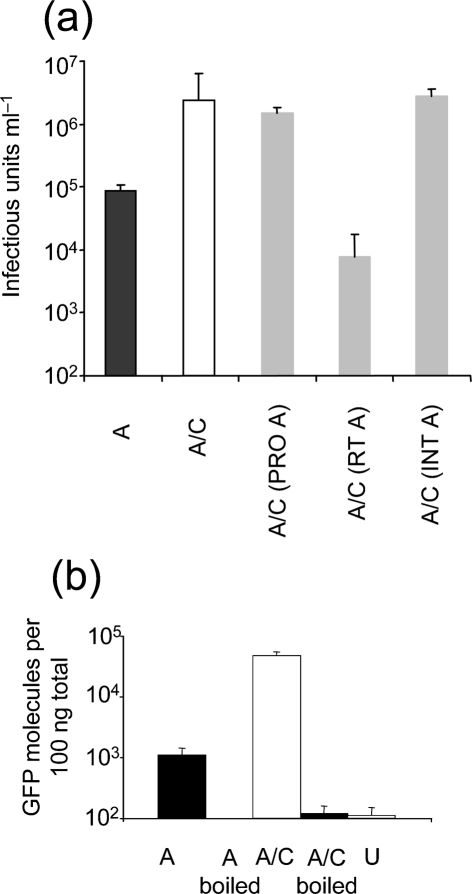
PERV A/C has more efficient reverse transcription than PERV A. (a) VSV-G pseudotypes of PERV A/C recombinant, PERV A or chimeric PERV A/C recombinants containing the protease, reverse transcriptase or integrase sequences of PERV A [PERV A/C (PRO A); PERV A/C (RT A); PERV A/C (INT A), respectively], were titrated and titres expressed as infectious units ml^−1^. Errors bars indicate sd derived from two experiments performed with independent virus stocks. (b) Equal doses of PERV A (black bars) and A/C recombinant (white bars) VSV-G pseudotypes were used to infect CRFK cells with a PERV A/C recombinant at an m.o.i. of 0.3 and the cells were incubated for 6 h. Values are expressed as the number of GFP-encoding DNA molecules per 100 ng total DNA. As a negative control, parallel virus samples were boiled for 5 min to inactivate the virus and then used to infect cells as shown. These samples gave background GFP levels, demonstrating that the GFP signal is due to viral reverse transcription. DNA prepared from uninfected cells was also subject to QPCR as a further negative control (grey bar) (U). Error bars indicate sd of duplicate samples and the data are representative of three replicates.

Next, we sought to map the PERV A protein responsible for the defect in infectivity. We made VSV-G pseudotypes using chimeric PERV A/C gag–pol, encoding individual PERV A proteins. Pseudotypes encoding PERV A protease or PERV A integrase were as infectious as PERV A/C (Fig. 3a[Fig f3]). However, PERV A/C encoding PERV A reverse transcriptase was one to two orders of magnitude less infectious, suggesting that PERV-A reverse transcriptase is less efficient, and that reduced DNA synthesis leads to reduced infectivity. To confirm the role for reverse transcriptase we tested whether the PERV A/C recombinant was better able to reverse transcribe than PERV A in target cells. We infected cells with equal doses of PERV A and PERV A/C recombinant for 6 h, purified total DNA and assayed for products of reverse transcription by Taqman QPCR, as described previously (Besnier *et al.*, 2002; Towers *et al.*, 1999). Data are presented as copies of reverse transcriptase (GFP) product per 100 ng total DNA (Fig. 3b[Fig f3]). This experiment shows that, indeed, the efficiency of PERV A/C reverse transcription is one to two orders of magnitude greater than PERV A. The increase in reverse transcriptase efficiency leads to an increase in VSV-G pseudotype infectivity by around the same magnitude (Figs 1a[Fig f1] and 3a[Fig f3]). The reverse transcriptase region of PERV A/C is derived from PERV C, and its amino acid sequence is identical to that of PERV C MSL (GenBank accession no. AF038600). PERV C reverse transcriptase is therefore more active than that of PERV A, possibly due to PERV C being around five million years younger than PERV A (Bartosch *et al.*, 2004; Niebert & Tonjes, 2005).

## DISCUSSION

It appears that PERVs, and perhaps gammaretroviruses in general, are insensitive to restriction by TRIM5*α*. This is surprising given the broad antiviral activity of some TRIMs against distantly related lentiviruses. For example, bovine TRIM5*α* restricts all the lentiviruses tested against it, including HIV-1, HIV-2, SIVmac, feline immunodeficiency virus and EIAV (Si *et al.*, 2006; Ylinen *et al.*, 2006). Moreover, rabbit TRIM5*α* restricts all but SIVmac (Schaller *et al.*, 2007) with the only viruses appearing to be insensitive to these two non-primate TRIM5*α*s being MLV-B and the two PERVs described herein. Indeed, MLV-B and the two PERVs are not sensitive to any of the TRIM5*α*s tested. Whilst it is clear that these studies are limited by the relatively small number of viruses they employ, we believe that their diversity is broad enough to demonstrate that gammaretroviruses are significantly less sensitive to restriction by TRIM5*α* molecules. A recent study has suggested that human TRIM5*α* protected humans from a gammaretrovirus found endogenized in the chimpanzee genome referred to as ptERV (Kaiser *et al.*, 2007). Whilst this theory seems reasonable, it is somewhat undermined by the observation that neither human nor chimpanzee TRIM5*α*s restrict MLVs bearing ptERV capsids (Perez-Caballero *et al.*, 2008). This more recent observation suggests that the constructs used by Kaiser *et al.*, which were derived from calculated consensus sequence, do not represent the behaviour of the ptERV virus.

The reason for poor sensitivity of gammaretroviruses to TRIM5*α* may lie in the capsid structure. Intriguingly, the region of the capsid shown to influence primate lentiviral sensitivity to TRIM5*α*, referred to as the cyclophilin A-binding loop, is missing in the MLV capsid, although the rest of the N-terminal capsid structure is highly conserved (Mortuza *et al.*, 2004). As changes in the cyclophilin-binding loop affect primate lentiviral sensitivity to restriction by TRIM5*α* (Berthoux *et al.*, 2005; Keckesova *et al.*, 2006; Lin & Emerman, 2008; Stremlau *et al.*, 2006b; Ylinen *et al.*, 2005), we speculate that this structure in gammaretroviruses has contributed to their general insensitivity to TRIM5*α*. It is striking that MLV-N is the only MLV shown to be restricted by TRIM5*α* (Hatziioannou *et al.*, 2004; Keckesova *et al.*, 2004; Perron *et al.*, 2004; Si *et al.*, 2006; Song *et al.*, 2005; Yap *et al.*, 2004; Ylinen *et al.*, 2005, 2006). MLV-N is essentially a point mutant of MLV-B and we suspect that the E110 to R MLV-B capsid change was selected by evolutionary pressure from the murine antiviral Fv1 N, giving an advantage in Fv1 B-encoding mice but rendering it rather sensitive to restriction by TRIM5*α*.

The experiments performed here have made use of VSV-G pseudotyped retroviral vectors produced in human 293T cells and overexpression of TRIM5*α* proteins in CRFK cells. It is therefore possible that this experimental system might have influenced the results. However, this system has been very helpful in determining TRIM5*α* sensitivities in the past. Furthermore, it has been consistent with what we know of tropism of retroviruses *in vivo*, for example the lack of HIV-1 replication in monkeys, which can be bypassed, at least *in vitro*, by obviating TRIM5*α* and APOBEC3G restriction (Hatziioannou *et al.*, 2006). In our view, overexpression of TRIM5*α* *in vitro* is unlikely to lead to specificity artefacts, on the basis that TRIM5*α* is strongly induced by interferon and therefore protein levels are likely to be high during viral infection *in vivo* (Asaoka *et al.*, 2005; Sakuma *et al.*, 2007). Overexpression experiments are therefore a sensitive and relevant test of restriction sensitivity, although reduction of TRIM5*α* expression in relevant cell types and demonstration that PERV titres are not significantly affected would also augment the experiments described here.

**Fig. 4. f4:**
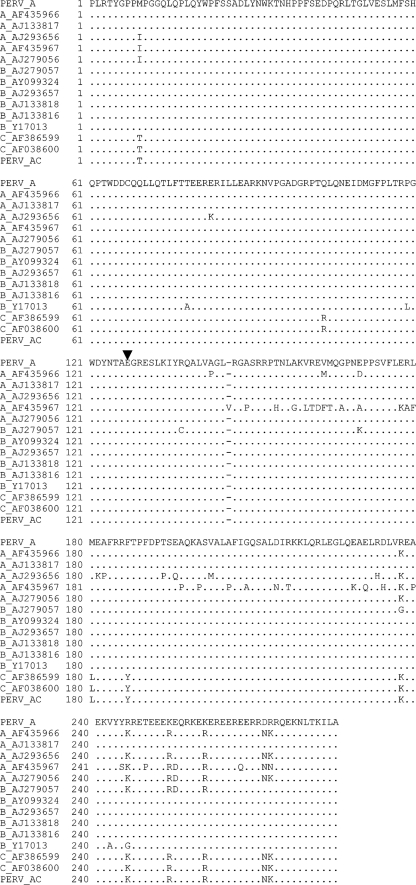
Alignment of capsid sequences from 15 PERVs indicates a high level of conservation. Sequences were retrieved and aligned using DNADynamo (Bluetractor Software) and Se-Al (Rambaut, 1996). GenBank accession numbers are shown, as is the classification into groups A, B or C according to Takeuchi *et al.* (1998). The PERV A and PERV A/C recombinants used in this study are included. The arrowhead indicates the PERV CA residue homologous to position CA 110 in MLV that influences sensitivity to restriction by TRIM5*α*. Dots indicate conserved residues.

We conclude that the two PERVs are not significantly restricted by any of the TRIM5*α* molecules tested (Fig. 1a[Fig f1]). The strongest restriction is threefold, by human TRIM5*α*, which is about the same magnitude that HIV-1 is restricted by human TRIM5*α* when overexpressed (Hatziioannou *et al.*, 2003; Newman *et al.*, 2006; Stremlau *et al.*, 2004; Yap *et al.*, 2004), indicating that it is unlikely to act as a barrier to PERV cross-species transmission. The two PERV sequences tested are likely to be representative of PERV classes A, B and C on the basis that the capsid sequences of these viruses are closely related (Fig. 4). Not only are they highly conserved, but the residue corresponding to MLV CA 110 (indicated by an arrowhead in Fig 4) known to influence MLV sensitivity to TRIM5*α* (Perron *et al.*, 2004; Towers *et al.*, 2000) is a conserved glutamate as it is in unrestricted MLV-B. This class of PERVs in general are therefore unlikely to be restricted by mammalian TRIM5*α* molecules. It is clear, however, that TRIM5*α* is not the only barrier to species-specific retroviral infection. Human APOBEC3G strongly restricts PERVs (Jonsson *et al.*, 2007) and human tetherin is likely to restrict PERVs, given that it restricts closely related MLVs (Neil *et al.*, 2008). There are also many other human TRIM proteins that may restrict PERVs (Nisole *et al.*, 2005). Our data therefore merely consider a single aspect of species barriers to zoonosis, which are likely to be complex and mediated by a large arsenal of species-specific antiviral proteins. Having said that, MLVs do appear to be particularly successful in transmitting between species, as illustrated by the diversity of gammaretroviral sequences in mammalian genomes, suggesting that they might be well adapted to avoiding restriction (Martin *et al.*, 1999, 2003).

We assume that the higher reverse transcriptase activity of the recombinant virus is due to its acquiring a PERV C reverse transcriptase sequence that has been inserted into the pig genome around five million years more recently than the PERV A sequence (Niebert & Tonjes, 2005). The more recent endogenization of the PERV C means that its reverse transcriptase sequence has been subject to fewer deleterious mutations, leading to higher activity. The apparent ease with which the relatively less infectious PERV A can acquire PERV C sequences to become highly infectious illustrates the plasticity of retroviruses and underlines the risk posed by introducing them into immunosuppressed individuals during xenotransplantation. This, and their apparent insensitivity to the important species barrier provided by TRIM5*α*, underscores the need to consider the possibility of zoonotic transmission occurring in the context of pig to human xenotransplantation.
